# Distinct Effects of Chronic Dopaminergic Stimulation on Hippocampal Neurogenesis and Striatal Doublecortin Expression in Adult Mice

**DOI:** 10.3389/fnins.2016.00077

**Published:** 2016-03-11

**Authors:** Rachele Salvi, Tobias Steigleder, Johannes C. M. Schlachetzki, Elisabeth Waldmann, Stefan Schwab, Beate Winner, Jürgen Winkler, Zacharias Kohl

**Affiliations:** ^1^Department of Molecular Neurology, Friedrich-Alexander University Erlangen-NürnbergErlangen, Germany; ^2^Department of Neurology, Friedrich-Alexander University Erlangen-NürnbergErlangen, Germany; ^3^Department of Palliative Medicine, Friedrich-Alexander University Erlangen-NürnbergErlangen, Germany; ^4^Department of Cellular and Molecular Medicine, University of CaliforniaSan Diego, CA, USA; ^5^Department of Medical Informatics, Biometry and Epidemiology, Friedrich-Alexander University Erlangen-NürnbergErlangen, Germany; ^6^IZKF Junior Research Group III and BMBF Research Group Neuroscience, Friedrich-Alexander University Erlangen-NürnbergErlangen, Germany

**Keywords:** adult neurogenesis, dentate gyrus, striatum, dopamine agonist, dopamine receptor, neuroblast, doublecortin, Parkinson's disease

## Abstract

While adult neurogenesis is considered to be restricted to the hippocampal dentate gyrus (DG) and the subventricular zone (SVZ), recent studies in humans and rodents provide evidence for newly generated neurons in regions generally considered as non-neurogenic, e.g., the striatum. Stimulating dopaminergic neurotransmission has the potential to enhance adult neurogenesis in the SVZ and the DG most likely via D_2_/D_3_ dopamine (DA) receptors. Here, we investigated the effect of two distinct preferential D_2_/D_3_ DA agonists, Pramipexole (PPX), and Ropinirole (ROP), on adult neurogenesis in the hippocampus and striatum of adult naïve mice. To determine newly generated cells in the DG incorporating 5-bromo-2′-deoxyuridine (BrdU) a proliferation paradigm was performed in which two BrdU injections (100 mg/kg) were applied intraperitoneally within 12 h after a 14-days-DA agonist treatment. Interestingly, PPX, but not ROP significantly enhanced the proliferation in the DG by 42% compared to phosphate buffered saline (PBS)-injected control mice. To analyze the proportion of newly generated cells differentiating into mature neurons, we quantified cells co-expressing BrdU and Neuronal Nuclei (NeuN) 32 days after the last of five BrdU injections (50 mg/kg) applied at the beginning of 14-days DA agonist or PBS administration. Again, PPX only enhanced neurogenesis in the DG significantly compared to ROP- and PBS-injected mice. Moreover, we explored the pro-neurogenic effect of both DA agonists in the striatum by quantifying neuroblasts expressing doublecortin (DCX) in the entire striatum, as well as in the dorsal and ventral sub-regions separately. We observed a significantly higher number of DCX^+^ neuroblasts in the dorsal compared to the ventral sub-region of the striatum in PPX-injected mice. These results suggest that the stimulation of hippocampal and dorsal striatal neurogenesis may be up-regulated by PPX. The increased generation of neural cells, both in constitutively active and quiescent neurogenic niches, might be related to the proportional higher D_3_ receptor affinity of PPX, non-dopaminergic effects of PPX, or altered motor behavior.

## Introduction

The generation of new neurons in the adult forebrain persists in the subgranular zone (SGZ) of the dentate gyrus (DG) and the subventricular zone (SVZ) of the lateral ventricles throughout the mammalian lifespan (Sanai et al., 2004; Ming and Song, 2005; Spalding et al., 2013), including non-human primates and humans (Eriksson et al., 1998; Ngwenya et al., 2006; Jabes et al., 2010; Spalding et al., 2013). Several extrinsic factors regulate proliferation and survival of neural precursor cells (NPCs) such as growth factors, hormones, and neurotransmitters in the “classical” constitutively active neurogenic niches (Brezun and Daszuta, 1999; Kulkarni et al., 2002). In this regard, the neurotransmitter dopamine (DA) plays a pivotal role (Winner et al., 2006; Berg et al., 2011) since dopaminergic fibers directly target hippocampal and SVZ NPCs (Hoglinger et al., 2004). Dopaminergic neurons in the midbrain, particularly from the substantia nigra pars compacta (SNc) and the ventral tegmental area (VTA) innervate the hippocampal formation, as well as the striatum (Bjorklund and Dunnett, 2007; Hoglinger et al., 2014). The SGZ is mainly targeted by dopaminergic projections of the caudal SNc (Hoglinger et al., 2004). In rodents, neurons of the SNc project toward the dorsal and lateral striatum in a topographically ordered medial-to-lateral arrangement forming the ascending nigrostriatal pathway (Perrone-Capano and Di Porzio, 2000). The previously defined non-neurogenic region, the striatum, possesses the ability to generate neuroblasts in response to cerebral ischemic stroke or traumatic injury and in corresponding animals models (Luzzati et al., 2011; Nato et al., 2015). Furthermore, striatal neurogenesis has also been observed in adult non-human primates, such as the squirrel monkey (Bedard et al., 2002). An indication for the generation of new neurons in the striatum also derives from a study in humans using a birth dating approach based on the incorporation of nuclear-bomb-test-derived ^14^C in the DNA of proliferating cells. Strikingly, a postnatal turnover of cells was observed in the human striatum post-mortem (Ernst et al., 2014).

Adult hippocampal neurogenesis is severely impaired in neurodegenerative diseases, in particular Parkinson's disease (PD), the second most common neurodegenerative disorder (Maj et al., 1997). One of the well-known hallmarks in PD is the loss of dopaminergic neurons in the SNc with the consecutive reduction of dopaminergic projections to the DG and the striatum (Bernheimer et al., 1973; Hoglinger et al., 2004). Although, the current treatment in PD is mainly constituted by levodopa and/or DA agonists to alleviate the diminished dopaminergic tone within the striatum, a better understanding of the micro-environmental signals regulating the generation of NPCs will provide the possibility to regionally increase the neural pool in the hippocampal formation and possibly in quiescent neurogenic areas like the striatum. DA agonists act by binding to different subsets of postsynaptic DA receptors classified into two groups based on their intracellular signaling properties, the D_1_- and the D_2_-like family. D_1_ and D_5_ receptors, belonging to the D_1_-like family, are coupled to G-proteins and thereby enhancing cyclic adenosine monophosphate (cAMP) levels, whereas D_2_, D_3_, and D_4_ classified as D_2_-like receptor, exert an opposite effect on cAMP resulting in decreased protein kinase A (PKA) activity (Missale et al., 1998). Pramipexole (PPX) is a D_2_-like selective, non-ergolinic DA agonist with 5- to 7-fold higher affinity selectivity for the D_3_ receptor compared to the D_2_ receptor and minimal activity on the D_4_ receptor (Mierau et al., 1995; Dooley and Markham, 1998). Ropinirole (ROP), another non-ergolinic DA agonist, has also a selectivity for D_2_-like family receptors, but exhibit less specificity for the D_3_ receptor in comparison to PPX (Tanaka et al., 2001). In rodents D_2_-like family receptors are anatomically distributed in telencephalic regions receiving dopaminergic afferents from the VTA (A10), such as the hippocampus and the whole striatum as observed by *in situ* hybridization and by qPCR analysis of RNA extracted from hippocampal and striatal regions (Sokoloff et al., 1990; Bouthenet et al., 1991; Mu et al., 2011).

Several *in vivo* studies focused on dopaminergic stimulation of adult neurogenesis within the SVZ or the SGZ in rodents with dopaminergic lesions (Winner et al., 2006; Chiu et al., 2015). Initially, there was evidence that levodopa restores proliferation of NPCs within the SVZ after 6-hydroxydopamine (6-OHDA) lesioning (Hoglinger et al., 2004). Furthermore, the proliferation of NPCs was reduced in the SVZ of 6-OHDA-lesioned rats (Winner et al., 2006), and consequently, PPX administration induced the proliferation of NPCs in the SVZ of 6-OHDA lesioned rats (Winner et al., 2009). Recently, treatment of levodopa and PPX restored decreased neurogenesis in the DG and periglomerular layer of the olfactory bulb in mice with bilateral intra-nigral 6-OHDA lesions (Chiu et al., 2015). Since these studies were performed in lesioned animals only, we explored the effects of the DA agonists, PPX and ROP, frequently used for the treatment in PD patients, on adult neurogenesis in the hippocampal SGZ and striatum of adult naïve mice.

## Materials and methods

### Animals

Naïve female C57BL/6 mice aged 3 months (obtained from Charles River Laboratories International, Inc.) were housed in a 12 h light/12 h dark cycle and had free access to food and water. All experiments were carried out in accordance with the European Communities Council Directive of November, 24th 1986 (86/609/ EEC) and were approved by the local governmental commission for animal health.

### Experimental design

Proliferation of NPCs and the survival of newly generated neurons in the DG were analyzed using three weight- and age-matched groups of animals [proliferation group: phosphate buffered saline (PBS), *n* = 5; PPX and ROP, *n* = 6; survival group: PBS, *n* = 5; PPX and ROP, *n* = 7]. For both designs, PPX, ROP, or PBS was administered by intraperitoneal injections (i.p., dissolved in 100 μl PBS) once per day for 14 consecutive days: animals received either PPX 0.3 mg/kg or ROP 3.0 mg/kg; the control animals were injected with 0.5% PBS only. The dose selection for PPX and ROP was based on previous studies where PPX treatment in a dose range between 0.1 and 1 mg/kg for up to 2 weeks was able to restore lesion-induced dopaminergic deficits in mice on a functional, biochemical, and structural level (Anderson et al., 2001; Jabes et al., 2010). Furthermore, ROP treatment for up to 1 week with doses between 0.5 and 3 mg/kg attenuated lesion-induced dopaminergic deficits in mice (Iida et al., 1999; Park et al., 2013). In addition, we referred to the levodopa equivalent dose (LED) representing an estimation of the DA agonist dose able to produce a similar antiparkinsonian effect as 100 mg of levodopa in humans. The standardized LED for PPX and ROP are 1 and 5 mg, respectively (Yamada et al., 1990). The 2-week treatment period with DA agonists was also based on previous studies reporting chronic DA agonist administration being more effective in enhancing adult neurogenesis than acute administration (Winner et al., 2009; Onoue et al., 2014; Takamura et al., 2014).

To label proliferating cells in the forebrain, 5-bromo-2′-deoxyuridine (BrdU) was injected i.p. twice on day 15 (100 mg/kg body weight; **Figure 2A**, proliferation paradigm). At day 15, animals were deeply anesthetized and transcardially perfused with 4% paraformaldehyde (PFA; Sigma) in 100 mM phosphate buffer (PB), pH 7.4. In order to detect the survival of newly generated cells in the DG, BrdU was administered once daily for the first 5 days (50 mg/kg, given in a volume of 100 μl) of the 14-days DA agonist treatment in a second cohort of mice, and after 2 weeks animals were perfused at day 37 (**Figure 3A**, survival paradigm).

### Tissue processing

Dissected brains were post-fixed in 4% PFA/PBS for 24 h, placed in a solution of 30% sucrose in PBS and cut into 40-μm coronal and sagittal sections using a sliding microtome (Leica, Germany) on dry ice. The sections were stored in cryoprotectant (ethylene glycol, glycerol, 0.1 M PB pH 7.4, 1:1:2 by volume) at −20°C until further processing for immunohistochemistry or -fluorescence.

### Immunohistochemistry and -fluorescence

Immunostainings were performed as previously described (Kohl et al., 2012). In order to detect BrdU, tissues were pre-treated with formamide and HCl in order to denature DNA. Free-floating sections in Tris-buffered saline (TBS: 0.15 M NaCl, 0.1 M Tris-HCl, pH 7.5) were treated with 0.6% H_2_O_2_ for 30 min. Following several washes in TBS, sections were blocked in 3% donkey serum and 0.1% Triton-X100 (Sigma) diluted in TBS for 1 h and incubated with primary antibodies in blocking solution overnight at 4°C. The primary antibodies used were monoclonal rat anti-BrdU (1:500, AbD Serotec, Oxford, UK), monoclonal mouse anti-Neuronal Nuclei (NeuN; 1:500, Millipore, Billerica, MA, USA), and polyclonal goat anti-DCX (1:250, Santa Cruz Biotechnology, CA, USA). For immunohistochemistry, tissues were treated with biotin-conjugated species-specific secondary antibodies followed by incubation with avidin-biotin-peroxidase complex (1:100) and 3, 3′-diaminobenzidine (DAB) substrate (both Vector Laboratories, Burlingame, CA, USA). For immunofluorescence, donkey-derived anti-mouse and anti-rat secondary antibodies were used conjugated with Alexa-568 and Alexa-488 or biotin (1:500, Dianova, Hamburg, Germany), respectively (all 1:1000, Invitrogen, Carlsbad, CA, USA). For all antibodies, control stainings without primary antibody showed no signal.

### Counting procedures

**Figure 1:**
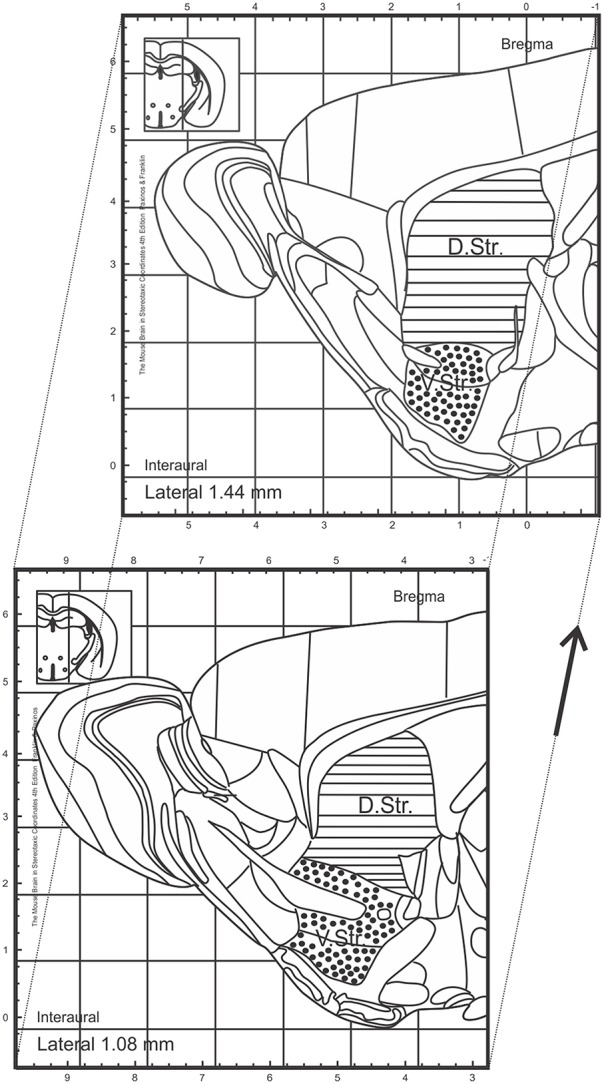
**Topographical map of sagittal sections of adult mice adopted from Franklin and Paxinos (2013)**. Regions analyzed within this study are marked and include the dorsal and ventral striatum. Sections analyzed between interaural lateral 1.44 and 1.08 mm. D.Str., dorsal striatum; V.Str., ventral striatum.

Slides were blind-coded and all counting procedures were performed on 40-μm sagittal sections. Every 6th section (240-μm interval) was selected and processed for immunohistochemistry. To analyze the number of BrdU^+^ cells in the granule cell layer (GCL) of the DG, BrdU-labeled cells were exhaustively counted on each section excluding the uppermost focal plane (exclusion plane) and the obtained values multiplied by 6, as an estimation of the total number of BrdU^+^ cells, both for the proliferation and survival paradigm (Williams and Rakic, 1988). All counting procedures and measurements of reference DG volumes (measured in mm^3^) were conducted on a light/fluorescence microscope (Zeiss AxioImager M2, Göttingen, Germany) equipped with a semi-automatic stereology system (Stereoinvestigator, MicroBrightField, Colchester, VT, USA) as previously described (Kohl et al., 2012). The subsequent densities of BrdU^+^ cells were calculated by dividing the number by the DG volume for each animal. To quantify the number of DCX^+^ neuroblasts in the entire striatum and consequently in the dorsal and ventral sub-regions, we used sections at the following coordinates: interaural lateral 1.44 and 1.08 mm. By using this approach, the boundaries between the dorsal and ventral striatum are anatomically well-defined by the anterior commissure (see Figure 1, adopted from Franklin and Paxinos, 2013). The identical procedures as above described were applied for the assessment of DCX^+^ cell numbers and striatal volumes. All bright-field images were obtained using the same microscope, as previously described.

To estimate the differentiation into a neuronal phenotype, every 12th section was stained for BrdU and NeuN by immunofluorescence and examined using a confocal laser microscope (ZEISS LSM780, Göttingen, Germany) equipped with a 40x PL APO oil objective and a pinhole setting that corresponds to a focal plane of 2 μm or less. In the DG, 40 to 50 BrdU^+^ cells from each animal were analyzed, randomly selected, and examined by moving through the z-axis of each cell in order to exclude false double labeling. BrdU^+^ cells were counted (newborn cells) and cells positive for both BrdU and NeuN (BrdU^+^/NeuN^+^ double-positive cells, newborn neurons) were assessed. The ratio of BrdU^+^/NeuN^+^ double positive cells by BrdU^+^ cells was determined. Applying the ratio of BrdU^+^/NeuN^+^ cells to the density of BrdU^+^ cells the number of newborn neurons was calculated. Fluorescent images were obtained using the identical confocal laser microscope (40x APO objective).

### Statistical analysis

All values are expressed as mean ± standard error of the mean (SEM). Statistical analysis was performed using one-way ANOVA comparisons between treatment groups followed by Tukey's *post-hoc* analysis (Prism 5; GraphPad, San Diego, CA, USA). For the analysis of DCX^+^ neuroblasts in the sub-regions of the striatum, we used a classical linear regression with a square root transformation of the dependent variable. Significance threshold was assumed at *p* < 0.05.

## Results

### PPX, but not ROP administration results in increased proliferation of NPCs in the hippocampal DG

**Figure 2:**
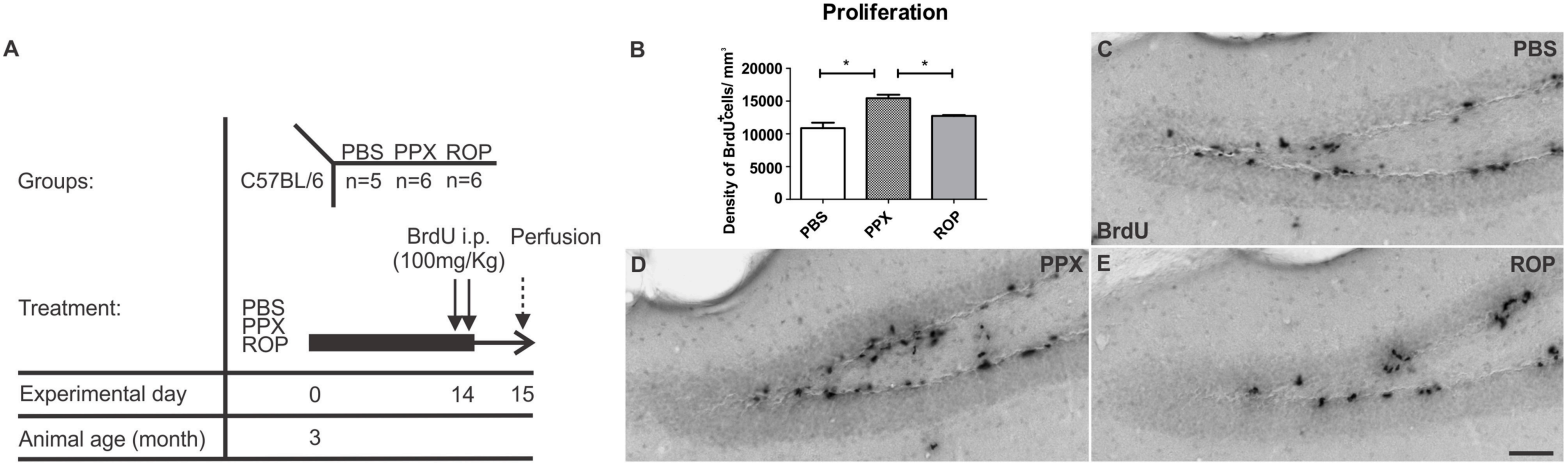
**Increased cell proliferation in the murine DG after chronic administration with PPX, but not after ROP treatment. (A)** Proliferation paradigm. 3-month-old C57BL/6 received either i.p. PPX, ROP or PBS for 14 days. At day 14, animals were i.p. injected with BrdU (100 mg/kg) twice to label proliferating cells. Mice were perfused at day 15. **(B)** Quantification of BrdU^+^ cells in the DG revealed an increased density of proliferating cells after PPX compared to ROP and PBS. Representative stainings of BrdU^+^ cells in the DG of PBS- **(C)**, PPX- **(D)**, and ROP-injected animals **(E)**. Error bars represent mean ± SEM. One-way ANOVA followed by Tukey's *post-hoc* test, ^*^p < 0.05. Scale bar: 50 μm **(C–E)**.

In order to investigate the effect of two different DA agonists on NPC proliferation in the adult DG, we compared the density of newborn cells between PPX-, ROP-, and PBS-injected animals. The groups were daily injected with PPX, ROP, or PBS for 14 days followed by two BrdU administrations at the same day prior to perfusion (Figure 2A). We quantified the number of BrdU^+^ cells in the DG and calculated their density. PPX significantly increased the number of BrdU^+^ cells compared to PBS-injected mice by 42% [PPX: 15421.3 ± 544.2, ROP: 12722.9 ± 129.4, PBS: 10864.2 ± 827.1, *F*_(2, 14)_ = 17.65, *p* < 0.001; Figures 2B–D; Table 1]. In contrast, there was no significant effect on the number of BrdU^+^ cells after administration of ROP (*p* > 0.05; Figures 2B,E; Table 1). In addition, there was no effect of both DA agonists on the DG volume [PPX: 0.183 ± 0.01, ROP: 0.174 ± 0.01, PBS: 0.198 ± 0.02, *F*_(2, 14)_ = 0.569, *p* > 0.05].

**Table 1:** **Summary of PPX and ROP treatment on hippocampal and striatal neurogenesis**.

	**PBS**	**PPX**	**ROP**
Hippocampal neurogenesis			
Proliferation DG: density of BrdU^+^ cells	10864.2 ± 827.1	15421.3 ± 544.2^###^^,^ ^++^	12722.9 ± 129.4
Newly generated neurons DG: density of BrdU^+^/NeuN^+^ cells	6014.0 ± 928.2	11468.0 ± 1254.0^##^^,^ ^+^	7369.0 ± 797.8
Striatal Neuroblasts			
DCX^+^ cells overall	134.4 ± 13.9	257.1 ± 63.1	157.7 ± 23.3
DCX^+^ dorsal striatum	103.2 ± 6.1	185.1 ± 41.6^#^	104.6 ± 16.5
DCX^+^ ventral striatum	31.2 ± 10.5	72.0 ± 23.6	53.1 ± 24.1

*PPX induced an increased number of dividing cells compared to ROP (by 21%) and PBS (by 42%) in the DG. A significant increased survival of newly generated neurons, identified by BrdU/NeuN co-labeling, was observed in the DG of PPX-injected mice in comparison to ROP (56%) and PBS (91%). PPX-injected mice showed a non-significant increase of DCX^+^ neuroblasts in the entire striatum compared to PBS- or ROP-injected mice. Data is shown as mean ± SEM. Comparison between groups in the DG and the entire striatum was performed by one-way ANOVA followed by Tukey's post-hoc test and in the dorsal and ventral striatum by using a classical linear regression. BrdU, 5-bromo-2′-deoxyuridine; NeuN, neuronal nuclei, DCX: doublecortin*.

### *Significance level p < 0.001 compared to PBS*;

## *Significance level p < 0.01 compared to PBS*;

# *Significance level p < 0.05 compared to PBS*;

++ *Significance level p < 0.01 compared to ROP*;

+ *Significance level p < 0.05 compared to ROP*.

### PPX doubles adult neurogenesis in the murine DG

**Figure 3:**
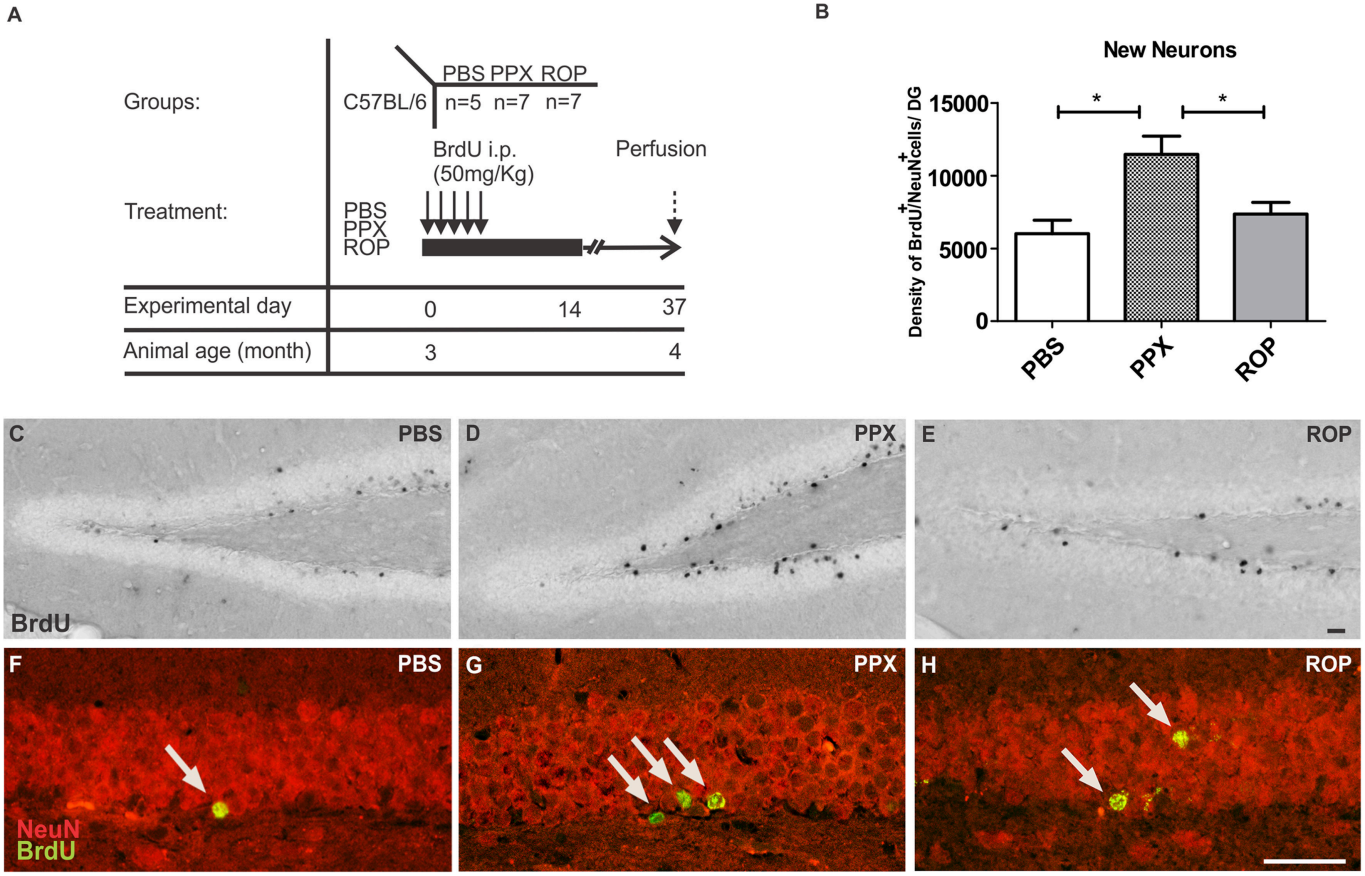
**PPX only increases the generation of new neurons in the murine DG. (A)** Survival paradigm. At the beginning, 3-month-old animals received PPX, ROP or PBS i.p. for 14 days. During the first 5 days, all mice were i.p. injected with BrdU (50 mg/kg). Animals were perfused 32 days after the last BrdU injection. **(B)** Hippocampal neurogenesis (calculated density of BrdU^+^/NeuN^+^ neurons) was increased after PPX compared to PBS. No change in DG neurogenesis was observed after ROP treatment. Representative stainings of BrdU^+^ in the DG of the hippocampus of PBS **(C)**, PPX **(D)**, and ROP **(E)** treated mice show a significant increase in PPX-injected animals compared to ROP- and PBS-injected controls. Confocal microscopy depicts double-labeled BrdU^+^ (green)/NeuN^+^ (red) neurons in the hippocampal DG of PBS **(F)**, PPX **(G)**, and ROP-injected mice **(H)**. Error bars represent mean ± SEM. One-way ANOVA followed by Tukey's *post-hoc* test, ^*^p < 0.05. Scale bars: 20 μm **(C–H)**.

Next, we analyzed whether 14 days of administration with PPX or ROP has a pro-neurogenic effect by determining the survival of newborn neurons in the DG 32 days after the last BrdU injection (Figures 3A,C–E). Therefore, we quantified the density of surviving new DG cells and determined the number of cells colabelling BrdU^+^ and the mature neuronal marker NeuN^+^ in the DG. Interestingly, we observed that the percentage of NeuN/BrdU double-labeled cells was significantly increased in PPX-injected mice compared to ROP and PBS [PPX: 88% ± 0.99, ROP: 73% ± 1.25, PBS: 70% ± 0.93, *F*_(2, 16)_ = 76.41, *p* < 0.05]. However, there was no effect of ROP compared to PBS-injected mice. Calculating the density of newborn neurons (BrdU^+^/NeuN^+^), PPX significantly enhanced hippocampal neurogenesis by 91% in comparison with PBS-injected mice [PPX: 11468.0 ± 1254.0, ROP: 7369.0 ± 797.8, PBS: 6014.0 ± 928.2, *F*_(2, 16)_ = 7.305, PPX vs. PBS *p* < 0.01, PPX vs. ROP *p* < 0.05; Figures 3B,F–H; Table 1]. In contrast, the administration of ROP did not result in an increased number of new neurons compared to PBS-injected mice (*p* > 0.05; Figure 3B). Again, DG volumes were not different between groups [PPX: 0.152 ± 0.01, ROP: 0.151 ± 0.01, PBS: 0.150 ± 0.008, *F*_(2, 16)_ = 0.008, *p* > 0.05].

### PPX predominantly promotes the generation of DCX^+^ neuroblasts in the dorsal striatum

**Figure 4:**
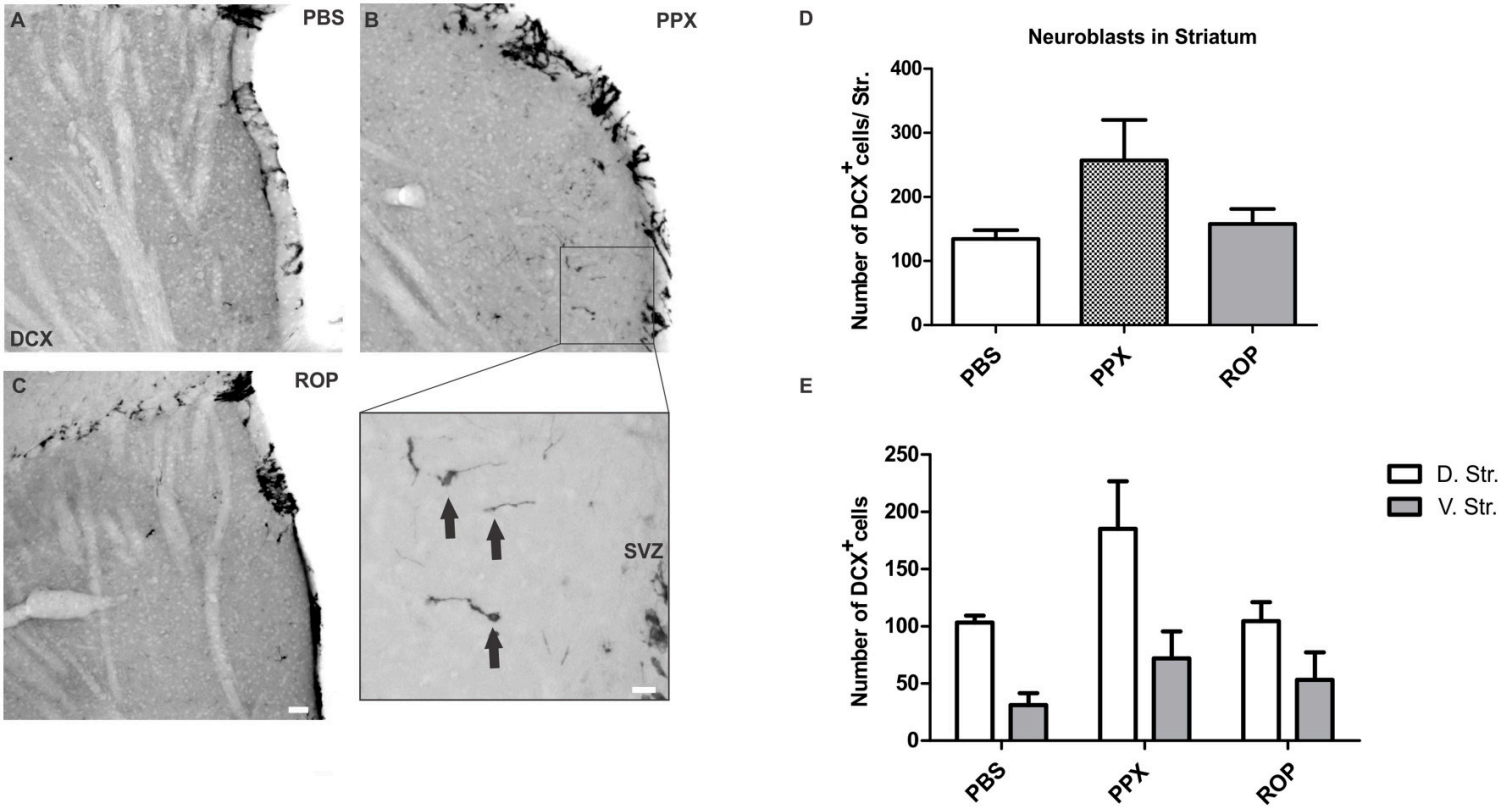
**PPX induces the generation of neuroblasts in the dorsal compared to the ventral striatum**. Representative stainings of DCX^+^ cells in the striatum showing a low number of neuroblasts in the PBS **(A)** and in the ROP group **(C)**, and a higher number in the PPX group **(B)**. Insert displays the region of interest with DCX^+^ cells at higher magnification. **(D–E)** Quantification of DCX^+^ neuroblasts in the entire striatum, and separately in the dorsal vs. ventral striatal sub-regions. No significant difference in the number of DCX^+^ cells was observed in the entire striatum for all groups (One-way ANOVA followed by Tukey's *post-hoc* test). In contrast, the analysis of striatal sub-regions by using a linear regression model revealed a significant effect of PPX, but not with ROP treatment. Please note that the regression model was calculated based on the square root transformed numbers of DCX neuroblasts, whereas the untransformed numbers are displayed in **E**. Error bars represent mean ± SEM. Scale bars: 50 μm **(A–C)**, 10 μm (insert). D.Str, dorsal striatum; V.Str, ventral striatum.

We further investigated whether chronic DA agonist treatment influences the number of neuroblasts within the striatum. We quantified the number of DCX^+^ cells in the striatum of animals from the survival paradigm. We observed that PPX and ROP had no significant effect on the total number of neuroblasts in the entire striatum compared with PBS-treated mice [PPX: 257.1 ± 63.1, ROP: 157.7 ± 23.3, PBS: 134.4 ± 13.9, *F*_(2, 16)_ = 2.237, *p* > 0.05; Figures 4A–D; Table 1]. To detect whether the dorsal striatum, being highly innervated by dopaminergic projections of the SNc, was affected by PPX or ROP administration to a greater extent in comparison to the ventral striatum, we analyzed the number of DCX^+^ cells in both sub-regions separately (Figure 1). To analyse the impact of PPX and ROP simultaneously on the subregions (dorsal striatum, PPX: 185.1 ± 41.6, ROP: 104.6 ± 16.5, PBS: 103.2 ± 6.1; ventral striatum, PPX: 72.0 ± 23.6, ROP: 53.1 ± 24. 1, PBS: 31.2 ± 10.5; Figure 4E), we fit a linear regression model on the number of DCX^+^ cells. To gain normality in the dependent variable a square root transformation was used. The effect of *region* was significant with a coefficient of −4.73 (*sd* = 1.00, *p* < 0.001, reference category: *dorsal*). In the categorical variable *treatment* (reference category: *PBS*) the effect of PPX was significant with a coefficient of 3.01 (*sd* = 1.28, *p* = 0.0246), while the effect of ROP was not significant with a coefficient of 0.606 (*sd* = 1.28, *p* = 0.639). The intercept of the model was 9.88. The volumes of the entire striatum did not differ significantly between groups [PPX: 3.065 ± 0.233, ROP: 2.788 ± 0.313, PBS: 2.962 ± 0.471, *F*_(2, 16)_ = 0.201, *p* > 0.05].

## Discussion

The chronic administration of the non-ergoline D_2_/D_3_-receptor agonist PPX is able to strongly stimulate cell proliferation as well as adult neurogenesis in the hippocampal DG of naive mice. In contrast, the D_2_/D_3_-receptor agonist ROP shows no pro-neurogenic effects, neither on cell proliferation nor on the survival of newly generated DG neurons. Moreover, PPX only resulted in an increased number of DCX^+^ neuroblasts in the dorsal striatum suggesting a specific PPX mediated effect in the mouse forebrain, both for constitutively active and quiescent neurogenic niches.

While increasing experimental evidence supports the pro-neurogenic effect of dopaminergic compounds such as levodopa or DA agonists for enhancing adult neurogenesis in the SVZ (Van Kampen et al., 2004; Borta and Hoglinger, 2007; Winner et al., 2009) their role in stimulating adult hippocampal neurogenesis is rather limited. In the present study we observed that mice injected with PPX (0.3 mg/kg) over 14 days showed an increase of proliferating cells by 42% in the hippocampal DG compared to PBS-injected controls. In contrast, chronic ROP administration (3 mg/kg) failed to stimulate hippocampal cell proliferation. So far, two studies examined the effect of DA agonists on cell proliferation in the hippocampus in naïve rats: Onoue and colleagues described a reduction of proliferating BrdU^+^ cells in the SGZ by 34% after administration of PPX for 14 days in Wistar rats using a higher dosage of PPX (1 mg/kg; Onoue et al., 2014; Table 2). Another study observed no changes in SGZ proliferation of Sprague Dawley rats following PPX administration for 21 days at two different dosages (0.3 or 1 mg/kg; Takamura et al., 2014). These apparent discrepancies between the present and previous studies in enhancing adult hippocampal neurogenesis after chronic PPX administration may be very likely explained by species differences related to distinct characteristics of the hippocampal neurogenic niche of mice and rats. Interestingly, there is a species difference in relation to the duration necessary for the maturation of new DG neurons (Snyder et al., 2009).

**Table 2:** **Overview of studies addressing the effects of DA agonists on hippocampal neurogenesis in naïve rodents**.

**Study**	**Species/background, age, gender**	**BrdU dosage/paradigm**	**Treatment**		**Proliferation**	**Survival**
Present study	C57BL/6 mice; 12 wks, female	2 × 100 mg/kg 24 h before perfusion or 5 × 50 mg/kg 32 d before perfusion	0.3 mg/kg PPX	14 d	1 d: 42% ⇑ (BrdU)	32 d: 91% ⇑ (BrdU/NeuN)
			3 mg/kg ROP	14 d	1 d: ⇔ (BrdU)	32 d: ⇔ (BrdU/NeuN)
Onoue et al., 2014	Wistar rat; male	4 × 75 mg/kg 6 h before perfusion	1 mg/kg PPX	14 d	1 d: ⇓ 34.2% (BrdU)	NA
Takamura et al., 2014	Sprague–Dawley rat; 7 wks, male	1 × 75 mg/kg 24 h before perfusion or 21 d before perfusion	0.3 or 1 mg/kg PPX	21 d	1 d: ⇔ (BrdU)	23 d: ⇔ (BrdU)
			10 or 30 mg/kg SKF38393	21 d	1d: ⇔ (BrdU)	23 d: 53% ⇑ (BrdU)

*Values indicate the percentage increase or decrease of BrdU^+^ and BrdU^+^/NeuN^+^ cells in the DG compared to the control groups of the respective studies. The age of the animals at the beginning of the experiments is given. ⇔; no effect vs. control, ⇑: increased effect vs. control; ⇓:: reduced effect vs. control. NA, not applicable, d, days, wks: weeks, BrdU, 5-bromo-2′-deoxyuridine; NeuN, neuronal nuclei*.

Besides analyzing the effect of PPX on the proliferation of hippocampal NPCs, the aim of this study was to determine whether these newly generated cells were able to differentiate toward a neuronal lineage. Here, we observed that PPX administration significantly enhanced the proportion of neuronal differentiation resulting in an increased density of newly generated neurons by 91% in the DG of adult naïve mice compared to PBS-injected controls. Again, ROP administration failed to enhance hippocampal neurogenesis. The present effect of PPX on the survival and differentiation of newly generated neurons in the DG is in contrast to the previous study by Takamura et al. in rats where PPX at two different dosages (0.3 and 1 mg/kg) failed to enhance the survival of newly generated cells (Takamura et al., 2014). However, the D3-receptor DA agonist SKF38393 increased the survival of newly generated cells by 53%, however without determining the cellular phenotype (Takamura et al., 2014). While the majority of previous findings are in contrast to the present study (see Table 2), it is very likely that in particular species differences may account for the divergent response to chronic DA agonist administration. This notion is supported by our previous study in 6-OHDA lesioned Sprague-Dawley rats, where chronic PPX administration was not able to enhance adult neurogenesis in the hippocampal DG (Winner et al., 2009). In support of this striking species difference in regard to the response to dopaminergic stimuli, a very recent study showed that PPX administration rescued an impaired DG neurogenesis in 6-OHDA lesioned mice (Chiu et al., 2015).

PPX, but not ROP had a strong pro-neurogenic effect on adult hippocampal neurogenesis in mice although both compounds are D_2_-like selective DA agonists. One possible explanation for the different efficacy between both DA agonists may be related to the distinct receptor binding profile of PPX with its proportional higher affinity to the D_3_ receptor. In addition, the chosen dosage for ROP may be too low, although recovery of dopaminergic parameters after lesioning has been observed in the range of 0.5–2.0 mg/kg in mice (Iida et al., 1999; Park et al., 2013). Furthermore, Li and colleagues demonstrated that PPX administered both at a low (0.1 mg/kg) or high dosage (0.5 mg/kg) showed neuroprotective effects in a murine PD model with an impaired ubiquitin-proteasome system (Li et al., 2010). Interestingly, pretreatment with the D_3_ receptor antagonist U99194 blocked the PPX-mediated neuroprotection implying a selectivity of PPX for the D_3_ subtype (Li et al., 2010). However, there is also evidence for the selectivity of ROP for the D_3_ subtype over human D_2_ and D_4_ receptors based on radio-ligand binding studies (Eden et al., 1991). Alternatively, the effect of DA agonists on proliferation and differentiation of hippocampal NPCs may be mediated by non-dopaminergic, pro-neurogenic mechanisms, e.g., the stimulation of distinct growth factors such as brain derived growth factor (BDNF) and glial cell derived neurotrophic factor (GDNF; Du et al., 2005), or altered physical activity (Yamada et al., 1990; Maj et al., 1997).

The recent discovery of adult neurogenesis in the human striatum and previously in the rodent striatum showed that the generation of new neurons takes place in regions generally considered as non-neurogenic (Bedard et al., 2002; Dayer et al., 2005; Ernst et al., 2014; Inta et al., 2015). In the present study, we addressed the question whether the DA agonists, PPX or ROP, promote the generation of DCX^+^ neuroblasts in the striatum of naïve adult C57BL/6 mice. Indeed, we observed DCX^+^ neuroblasts throughout the striatum possibly constituting an endogenous cellular pool with the potential to further differentiate into neurons under physiological conditions, in the context of a compromised striatal microenvironment or upon specific exogenous stimuli. Interestingly, the number of DCX^+^ neuroblasts was significantly higher in the dorsal compared to the ventral sub-region of the striatum in the PPX group only. This dorsal-ventral gradient may reflect the fact that the dorsal striatum receives the majority of dopaminergic projections from the SNc (Haber, 2014).

Previously, a 10-fold increase in the number of BrdU^+^ cells was observed in the dorsal striatum of adult Sprague-Dawley rats following the intraventricular administration of the D_3_-receptor agonist 7-hydroxy-N,N-di-n-propyl-2-aminotetralin (Van Kampen et al., 2004). However, systemic treatment with PPX both in 6-OHDA and PBS medial forebrain bundle injected rats did not increase the number of DCX^+^ neuroblasts in the dorsal striatum (Winner et al., 2009). At present, it is still an open question whether DCX^+^ neuroblasts or newly generated neurons within the striatum evade from the adjacent SVZ or are locally generated from an endogenous progenitor pool within the striatal parenchyma. Interestingly and similar to the post-mortem analysis in humans, we observed an increased number of DCX^+^ neuroblasts in the striatum adjacent to the SVZ in transgenic mouse and rat models of Huntington's disease (HD) suggesting that the damaged striatum may attract to some extent immature neuroblasts from the adjacent SVZ (Kohl et al., 2010; Kandasamy et al., 2015). However, it is important to note that the lesioned striatum in PD models even after stimulation with growth factors such as the epidermal growth factor or the fibroblast growth factor-2 failed to provide a sufficient stimulus for immature DCX^+^ neuroblasts to obtain a mature neuronal phenotype (Winner et al., 2008). More recently, a long-lasting inflammatory response within the striatum after lesioning was observed and may explain the failure to differentiate into mature neurons (Schlachetzki et al., 2014). Taken together, this study provides evidence that species and the specific strain largely matter when investigating effects of the generation of new neurons in neurogenic and non-neurogenic regions following compound treatment.

## Author contributions

RS and TS performed experiments, collected and analyzed data, interpreted results, and partially wrote the manuscript. EW performed statistical analyses and interpreted the data. JS and SS discussed and interpreted the data. BW conceived the study and planned experiments. JW and ZK conceived the study, planned experiments, interpreted the data and edited the entire manuscript.

### Conflict of interest statement

The authors declare that the research was conducted in the absence of any commercial or financial relationships that could be construed as a potential conflict of interest.

## Abbreviations

6-OHDA: 6-hydroxydopamine
BDNF: brain derived growth factor
BrdU: 5-bromo-2′-deoxyuridine
cAMP: cyclic adenosine monophosphate
d: days
DA: dopamine
DAB: 3,3′-diaminobenzidine
DCX: doublecortin
DG: dentate gyrus
D.Str.: dorsal striatum
GCL: granule cell layer
GDNF: glial cell derived neurotrophic factor
HD: Huntington's disease
i.p.: intraperitoneal
LED: levodopa equivalent dose
NA: not applicable
NeuN: neuronal nuclei
NPCs: neural precursor cells
PB: phosphate buffer
PBS: phosphate buffered saline
PD: Parkinson's disease
PFA: paraformaldehyde
PKA: protein kinase A
PPX: pramipexole
ROP: ropinirole
SEM: standard error of the mean
SGZ: subgranular zone
SNc: substantia nigra pars compacta
SVZ: subventricular zone
TBS: Tris-buffered saline
V.Str.: ventral striatum
VTA: ventral tegmental area
wks: weeks.
